# Fixed Points of Difference Operator of Meromorphic Functions

**DOI:** 10.1155/2014/103249

**Published:** 2014-01-19

**Authors:** Zhaojun Wu, Hongyan Xu

**Affiliations:** ^1^School of Mathematics and Statistics, Hubei University of Science and Technology, Xianning 437100, China; ^2^Department of Informatics and Engineering, Jingdezhen Ceramic Institute, Jingdezhen 333403, China

## Abstract

Let *f* be a transcendental meromorphic function of order less than one. The authors prove that the exact difference Δ*f* =(*z*+1) - *f* (*z*) has infinitely many fixed points, if a ∈ *ℂ* and ∞ are Borel exceptional values (or Nevanlinna deficiency values) of *f*. These results extend the related results obtained by Chen and Shon.

## 1. Introduction and Main Results

In this paper, we assume that the reader is familiar with the notations of frequency use in Nevanlinna theory (see [1–3]). Let *f*(*z*) be a meromorphic function in the complex plane *ℂ* and *a* ∈ *ℂ*. We use the notations *σ*(*f*) to denote the order of *f*(*z*), *λ*(*f*, *a*), and *λ*(1/*f*), respectively, to denote the exponent of convergence of zeros of *f*(*z*) − *a* and poles of *f*(*z*). Especially, if *a* = 0, we denote *λ*(*f*, 0) = *λ*(*f*). A point *z* ∈ *ℂ* is called as a fixed point of *f*(*z*) if *f*(*z*) = *z*. There is a considerable number of results on the fixed points for meromorphic functions in the plane; we refer the reader to Chuang and Yang [4]. It follows Chen and Shon [5]; we use the notation *τ*(*f*) to denote the exponent of convergence of fixed points of *f* that is defined as

(1) $$\begin{array}{c} \tau \left(f\right)=\underset{r\to \infty }{\mathrm{limsup}}\frac{\log N\left(r,1/\left(f-z\right)\right)}{\log r}. \end{array}$$

Let *f* be a transcendental meromorphic function in the complex plane *ℂ*. The exact differences Δ*f* are defined by Δ*f* = *f*(*z* + 1) − *f*(*z*).

Recently, there are a number of papers (including [6–16]) focusing on the differences analogues of Nevanlinna's theory and its application on the complex difference equations. For the fixed points of the difference operator Δ*f*, Chen and Shon have proved the following.

**Theorem A (see [17])**

Let *f* be a transcendental entire function of order of growth *σ*(*f*) = 1 and have infinitely many zeros with the exponent of convergence of zeros *λ*(*f*) < 1. Then Δ*f* has infinitely many zeros and infinitely many fixed points.

When the order of *f* is less than 1, Chen and Shon have proved the following.

**Theorem B (see [5])**

Let *f* be a transcendental meromorphic function of order of growth *σ*(*f*) ≤ 1. Suppose that *f* satisfies *λ*(1/*f*) < *λ*(*f*) < 1 or has infinitely many zeros (with *λ*(*f*) = 0) and finitely many poles. Then Δ*f* has infinitely many fixed points and satisfies the exponent of convergence of fixed points *τ*(Δ*f*) = *σ*(*f*).

A natural question is, letting *f* be a transcendental meromorphic function of order of growth *σ*(*f*) < 1, is there a similar result as that in Theorem B if *λ*(1/*f*) ≥ *λ*(*f*) or *f* has infinitely many zeros (with *λ*(*f*) = 0) and infinitely many poles?

In this paper, we will prove the following theorem to answer the question.

**Theorem 1 (main)**

Let *f* be a transcendental meromorphic function of order of growth *σ*(*f*) < 1 and *a* ∈ *ℂ*. Suppose that *f* satisfies *λ*(1/*f*) < *σ*(*f*) and *λ*(*f*, *a*) < *σ*(*f*). Then Δ*f* has infinitely many fixed points and satisfies the exponent of convergence of fixed points *τ*(Δ*f*) = *σ*(*f*).

From Theorem 1, we can get the following corollary.

**Corollary 2**

Let *f* be a transcendental meromorphic function of order of growth *σ*(*f*) < 1. Suppose that *f* satisfies *λ*(*f*) ≤ *λ*(1/*f*) < *σ*(*f*). Then Δ*f* has infinitely many fixed points and satisfies the exponent of convergence of fixed points *τ*(Δ*f*) = *σ*(*f*).

In Theorem 1, we suppose that *f* satisfies *λ*(1/*f*) < *σ*(*f*) and *λ*(*f*, *a*) < *σ*(*f*). That is to say *∞* and *a* are Borel exceptional values of *f*. If we suppose that *∞* and *a* are Nevanlinna deficiency values of *f*, is there a similar result as that in Theorem B? In the following, we give Theorem 3 to answer this question.

Let *f*(*z*) be a meromorphic function in the complex plane *ℂ* and *a* ∈ *ℂ*_*∞*_ = *ℂ* ∪ {*∞*}. Nevanlinna's deficiency of *f* with respect to *a* is defined by

(2) $$\begin{array}{c} \delta \left(a,f\right)=1-\underset{r\to \infty }{\mathrm{limsup}}\frac{N\left(r,1/\left(f-a\right)\right)}{T\left(r,f\right)}. \end{array}$$

If *a* = *∞*, then one should replace *N*(*r*, 1/(*f* − *a*)) in the above formula by *N*(*r*, *f*). If *δ*(*a*, *f*) > 0, then *a* is called a Nevanlinna deficiency value of *f*.

**Theorem 3 (main)**

Let *f* be a transcendental meromorphic function of order of growth *σ*(*f*) < 1 and *a* ∈ *ℂ*. Suppose that *f* satisfies *δ*(*∞*, *f*) = 1 and *a* is a Nevanlinna deficiency value of *f*. Then Δ*f* has infinitely many fixed points.

**Corollary 4**

Let *f* be a transcendental entire function of order of growth *σ*(*f*) < 1 and *a* ∈ *ℂ*. Suppose that *δ*(*a*, *f*) > 0. Then Δ*f* has infinitely many fixed points.

## 2. Some Lemmas

**Lemma 1 (lemma on the logarithmic derivative)**

Let *f*(*z*) be a meromorphic function. If the function *f*(*z*) has finite order, then

(3) $$\begin{array}{c} m\left(r,\frac{f^{\left(k\right)}}{f}\right)=O\left(\log r\right) \end{array}$$

holds for any positive integer *k*.

**Lemma 2 (see [18])**

Let *f*(*z*) be a meromorphic function with the exponent of convergence of poles *λ*(1/*f*) = *λ* < +*∞* and let *c* be a nonzero complex number. Then for each *ɛ* > 0, we have

(4) $$\begin{array}{c} N\left(r,f\left(z+c\right)\right)=N\left(r,f\right)+O\left(r^{\lambda -1+ɛ}\right)+O\left(\log r\right). \end{array}$$

**Lemma 3**

Let *f* be a transcendental meromorphic function of order of growth *σ*(*f*) < 1 and let *c* be a nonzero complex number. Then

(5) $$\begin{array}{c} N\left(r,f\left(z+c\right)\right)=N\left(r,f\right)+O\left(\log r\right). \end{array}$$

**Proof**

Since the order *σ*(*f*)≔*σ* < 1, then *λ*(1/*f*) = *λ* ≤ *σ* < 1. Therefore, for any 0 < *ɛ* < 1 − *σ*, it follows from Lemma 2 that

(6) N(r,f(z+c))=N(r,f)+O(rλ−1+ɛ)+O(log⁡⁡r)=N(r,f)+O(1)+O(log⁡⁡r).

That is,

(7) $$\begin{array}{c} N\left(r,f\left(z+c\right)\right)=N\left(r,f\right)+O\left(\log r\right). \end{array}$$

**Lemma 4 (see [6])**

Let *f* be a function transcendental and meromorphic in the plane which satisfies

(8) $$\begin{array}{c} \underset{r\to \infty }{\mathrm{liminf}}\frac{T\left(r,f\right)}{r}=0. \end{array}$$

Then Δ*f* is transcendental.

**Lemma 5**

Let *f* be a transcendental meromorphic function of order of growth *σ*(*f*) = *σ* < 1. Then Δ*f* is transcendental.

**Proof**

Since the order *σ*(*f*)≔*σ* < 1, then, for any positive *ɛ*(0 < *ɛ* < 1 − *σ*), there exists *R* > 0 such that for any *r* > *R* we have

(9) $$\begin{array}{c} T\left(r,f\right)\leq r^{\sigma +ɛ}. \end{array}$$

Therefore,

(10) $$\begin{array}{c} \underset{r\to \infty }{\mathrm{liminf}}\frac{T\left(r,f\right)}{r}=0. \end{array}$$

Lemma 5 follows Lemma 4.

**Lemma 6 (see [7])**

Let *f*(*z*) be a meromorphic function of finite order, then *σ*(Δ*f*) ≤ *σ*(*f*).

**Lemma 7 (see [7])**

Let *f* be a transcendental meromorphic function of order of growth *σ*(*f*) < 1. Then for any *ɛ* > 0 and any positive integer *k*, there exists a set *E* ⊂ (1, *∞*) that depends on *f* and has finite logarithmic measure, such that for all *z* satisfying |*z* | = *r* ∉ *E* ∪ [0,1] we have

(11) $$\begin{array}{c} \frac{\Delta^{k}f\left(z\right)}{f\left(z\right)}=\frac{f^{\left(k\right)}\left(z\right)}{f\left(z\right)}+O\left(r^{(k+1)(\sigma -1)+ɛ}\right). \end{array}$$

It is easy to derive the following lemma from Lemma 1 and Lemma 7.

**Lemma 8**

Let *f* be a transcendental meromorphic function of order of growth *σ*(*f*) < 1. Then for any positive integer *k* there exists a set *E* ⊂ (1, *∞*) that depends on *f* and has finite logarithmic measure, such that

(12) $$\begin{array}{c} m\left(r,\frac{\Delta^{k}f\left(z\right)}{f\left(z\right)}\right)=O\left(\log r\right),\;\;\;r\notin E. \end{array}$$

## 3. Proof of Theorems

**Proof**

Since

(13) $$\begin{array}{c} \frac{1}{f}=\frac{\Delta f}{zf}-\frac{z\Delta^{2}f-\Delta f}{zf}\frac{\Delta f-z}{z\Delta^{2}f-\Delta f}, \end{array}$$

then

(14) m(r,1f)≤m(r,Δfzf)+m(r,zΔ2f−Δfzf) +m(r,Δf−zzΔ2f−Δf)+O(1)≤2m(r,Δff)+m(r,Δ2ff) +m(r,Δf−zzΔ2f−Δf)+O(log⁡⁡r).

Applying the first fundamental theorem, we get

(15) m(r,1f)=T(r,f)−N(r,1f)+O(1),m(r,Δf−zzΔ2f−Δf)=m(r,zΔ2f−ΔfΔf−z) +N(r,zΔ2f−ΔfΔf−z) −N(r,Δf−zzΔ2f−Δf)+O(1)≤m(r,zΔ2f−ΔfΔf−z) +N(r,zΔ2f−ΔfΔf−z)+O(1).

Combining (14)-(15) we have

(16) T(r,f)≤N(r,1f)+2m(r,Δff)+m(r,Δ2ff) +m(r,zΔ2f−ΔfΔf−z) +N(r,zΔ2f−ΔfΔf−z)+O(log⁡⁡r)≤N(r,1f)+N(r,1Δf−z)+N(r,zΔ2f−Δf) +2m(r,Δff) +m(r,Δ2ff)+m(r,zΔ2f−ΔfΔf−z)+O(log⁡⁡r).

Since

(17) Δ2f=Δ(f(z+1)−f(z))=f(z+2)−2f(z+1)+f(z),Δ(Δf−z)=Δ(f(z+1)−f(z)−z)=f(z+2)−2f(z+1)+f(z)−1,

then, we can get

(18) zΔ2f−Δf=zf(z+2)−2zf(z+1)+zf(z)−f(z+1)+f(z).zΔ(Δf−z)−(Δf−z)=zf(z+2)−2zf(z+1)+zf(z)−f(z+1)+f(z).

Therefore,

(19) zΔ2f−ΔfΔf−z=zΔ(Δf−z)−(Δf−z)Δf−z=zΔ(Δf−z)Δf−z−1,

(20) N(r,zΔ2f−Δf)≤N(r,f(z+2))+N(r,f(z+1)) +N(r,f(z)).

Thus from Lemma 3 and (20), we deduce

(21) $$\begin{array}{c} \begin{array}{cc} N\left(r,z\Delta^{2}f-\Delta f\right)\leq 3N\left(r,f\left(z\right)\right)+O\left(\log r\right). & \end{array} \end{array}$$

By Lemmas 5 and 6, we know that Δ*f* − *z* is a transcendental meromorphic function of order of growth *σ*(Δ*f* − *z*) ≤ *σ*(*f*) < 1. It follows from Lemma 8 and (19) that there exists a set *E* ⊂ (1, *∞*) that has finite logarithmic measure, such that for any *r* ∉ *E* we have

(22) $$\begin{array}{c} m\left(r,\frac{\Delta f}{f}\right)=O\left(\log r\right),\\ m\left(r,\frac{\Delta^{2}f}{f}\right)=O\left(\log r\right),\\ m\left(r,\frac{z\Delta^{2}f-\Delta f}{\Delta f-z}\right)=O\left(\log r\right). \end{array}$$

From (16) and (21)-(22), we have

(23) T(r,f)≤3N(r,f)+N(r,1f)+N(r,1Δf−z) +O(log⁡⁡r), r∉E.

Denoting *g* ≡ *f* − *a* by (23) we derive,

(24) T(r,f)≤T(r,g)+O(1)≤3N(r,g)+N(r,1g)+N(r,1Δg−z) +O(log⁡⁡r)≤3N(r,f)+N(r,1f−a)+N(r,1Δf−z) +O(log⁡⁡r), r∉E.

### 3.1. The Rest of the Proof of Theorem 1

By Lemma 6, we know that *τ*(Δ*f*) ≤ *σ*(*f*). If *τ*(Δ*f*) < *σ*(*f*), by *λ*(1/*f*) < *σ*(*f*) and *λ*(*f*, *a*) < *σ*(*f*), there exists a number *η* < *σ*(*f*), such that for any sufficient  *r* we have

(25) $$\begin{array}{c} N\left(r,f\right)<r^{\eta },\;\;N\left(r,\frac{1}{f-a}\right)<r^{\eta },\\ N\left(r,\frac{1}{\Delta f-z}\right)<r^{\eta }. \end{array}$$

Combining (24) and (25), we can get a contradiction. Therefore, we have *τ*(Δ*f*) = *σ*(*f*).

### 3.2. The Rest of the Proof of Theorem 3

Since *δ*(*∞*, *f*) = 1, then *N*(*r*, *f*) = *o*(*T*(*r*, *f*)). By (24), we can get

(26) (1−o(1))T(r,f)≤N(r,1f−a)+N(r,1Δf−z) +O(log⁡⁡r), r∉E.

Since *δ*(*a*, *f*) > 0, then there is a positive number *θ* < 1 such that

(27) $$\begin{array}{c} N\left(r,\frac{1}{f-a}\right)<\theta T\left(r,f\right). \end{array}$$

If Δ*f* has only a finite number of fixed points, then from (26) and (27) we would have

(28) $$\begin{array}{c} \left(1-o\left(1\right)-\theta \right)T\left(r,f\right)\leq O\left(\log r\right),\;r\notin E. \end{array}$$

This contradicts *f* being transcendental. Therefore, Δ*f* has infinitely many fixed points.
